# Editorial: Oxidative Stress: How Has It Been Considered in the Design of New Drug Candidates for Neurodegenerative Diseases?

**DOI:** 10.3389/fphar.2020.609274

**Published:** 2020-12-09

**Authors:** Claudio Viegas, Carlos A. Manssour Fraga, Maria Emilia Sousa, Andrea Tarozzi

**Affiliations:** ^1^ Institute of Biomedical Sciences, Federal University of Rio de Janeiro, Rio de Janeiro, Brazil; ^2^ Faculty of Pharmacy, University of Porto, Porto, Portugal; ^3^ Department for Life Quality Studies, University of Bologna, Bologna, Italy; ^4^ Laboratory of Research in Medicinal Chemistry (PeQuiM), Federal University of Alfenas, Alfenas, Brazil

**Keywords:** oxidative stress, neurodegenerative diseases, Alzheirmer's disease, Parkinsion’s disease, Huntington (disease), amyotrofic lateral sclerosis, neurodenegerative disorders, neurodegenaration

Central Nervous System (CNS) disorders affect millions of people worldwide (Feigin et al., 2019). Despite their growing impact on health systems and the urgent need for new, safer, and more effective drugs, the Pharmaceutical Industries have avoided efforts to develop new candidates due to high costs and low success rates in the clinical phase (Seyhan, 2019), mainly because of the insufficient knowledge of the mechanisms underlying CNS dysfunctions (Gribkoff and Kaczmarek, 2017).

In this scenario, neurodegenerative diseases (NDs), i.e., those that result from loss of function and death of nerve cells in the brain or peripheral nervous system, such as Parkinson's disease (PD), Alzheimer's disease (AD), Huntington's disease (HD) and amyotrophic lateral sclerosis (ALS), deserve to be highlighted due to their increasing prevalence, most of the time dependent on age, and the absence of treatments capable of controlling and reversing the processes associated with their pathogenesis (Martin, 1999). Therefore, the need for a better understanding of the complex mechanisms associated with the genesis of NDs and the development of new therapeutically effective approaches become essential (Golde, 2009; Santiago et al., 2017).

Although the clinical and neuropathological aspects of these disorders are distinct and most have the formation of abnormal protein deposits as a crucial factor associated with their onset, all have a common and characteristic pattern of neuronal degeneration in anatomically or functionally related regions (Vadakkan, 2016). Moreover, oxidative stress (OS) is considered as a common key player in the etiology and progression of these NDs and, for this reason, it could be observed a significant increase of interest in searching antioxidant and also anti-inflammatory effects of diverse classes of natural and synthetic compounds as promising drug candidates for the treatment of NDs (Chen et al., 2012).

Abundant literature data suggest that OS may induce not only cellular and membrane damage, but also DNA repair system breakdown or mitochondrial disfunction, contributing to a complex network of events related to energy supply, neurodegeneration and aging, a phenomena observed in AD or PD, for example (Schieber and Chandel, 2014). Increased reactive oxygen species (ROS) levels is a prompt consequence of OS and it is usually related to down-regulation in several defense systems including antioxidant enzymes or endogenous small-molecule antioxidants (Sayre et al., 2001). In this context, several hypotheses have been recently proposed to explain the complexity and multifactorial pathogenesis of AD, including OS as not only a central player, but, perhaps, one of the main causative factors of NDs, unifying a series of other sequential or individual pathophysiological events (Singh et al., 2019). According to this consensus, oxidative damage in the brain of patients is resultant from excessive production of free radicals induced by fragments of insoluble and/or overproduced proteins, such as β-amyloid peptide, α-synuclein, tau and huntingtin, with functional alteration in the mitochondria, inadequate energy supply, production of inflammatory mediators and alteration of antioxidant defenses (Islam, 2016; Liu et al., 2017). Thus, the modulation of the cellular oxidative process should lead to a new concept in the design of drugs and, possibly, a new way of searching for more effective disease-modifying chemical entities, reinforcing the hope of at least real clinical relief, if healing is not yet possible (Ghosh et al., 2011; Rekatsina et al., 2020).

The collection of research articles and reviews that composed this Research Topic clearly demonstrate some advances to better understand the role of OS in the mechanisms associated with the genesis of NDs and the ability of some natural products to modulate these phenomena. In this context, the paper by Huang et al. (2020) describes the neuroprotective effects of α-cyperone, a terpene isolated from *Cyperus rotundus*, against hydrogen peroxide (H_2_O_2_)-induced OS and apoptosis in dopaminergic neuronal SH-SY5Y cells, used as models to study PD. Their findings indicated that α-cyperone, which is well-known to inhibit neuroinflammation, was able to decrease H_2_O_2_-induced death and production of ROS in SH-SY5Y cells, through a mechanism dependent on the activation of nuclear factor erythroid 2-related factor 2 (Nrf-2), which is a key transcription factor for the regulation of antioxidant proteins expression. This behavior makes this class of natural products attractive in the search for newer therapies for treatment of PD.

Mhillaj et al. (2019) described in their review the effects of the natural antioxidant products ferulic acid, resveratrol and *Ginkgo biloba* extracts on heme oxygenase/biliverdin reductase system, which could be responsible for producing OS in neurons under unbalanced redox conditions. Despite countless preclinical studies with these herbal products demonstrating their neuroprotective effects, the compiled results were not sufficiently indicative of the efficacy of these natural products for NDs treatment, mainly because most of them showed poor bioavailability in humans. On the contrary, the results highlight the importance of taking care with the use of these herbal-derived products due to their ability to promote drug interactions resulting from their inhibitory or up-regulatory actions on different CYP isoforms.

On the other hand, the paper of Zhou et al. (2019) described the anti-inflammatory effects of cryptotanshinone, a diterpene extracted from *Salvia miltiorrhiza*, in neuroinflammation models induced by lipopolysaccharide in BV-2 microglial cells. By modulating Nrf2/heme-oxygenase 1 signaling pathway cryptotanshinone attenuates the upregulated expression of several proinflammatory proteins, such as inducible nitric oxide synthase, cyclooxygenase 2, NOD-like receptor pyrin domain-containing-3 (NLRP3), and it also reduces the increased release of pro-inflammatory cytokines, such as interleukin-1β, interleukin-6 and tumor necrosis factor-α. These results were important to elucidate the mechanisms associated with the anti-inflammatory effects of cryptotanshinone, opening new horizons for the possibility of research and development of new analogues for the therapy of NDs related with microglial cell activation.

Moreover, the actions promoted by the commonly found natural product luteolin on Nrf-2 translocation to the nucleus and the improvement of the expression of antioxidant proteins were the subject of the paper by Tan et al. (2020). Their findings indicated the beneficial neuroprotective effects of this flavonoid in intracerebral hemorrhage induced secondary brain injury, through the enhancement of autophagy and anti-oxidative processes dependent on activation of the p62-Keap1-Nrf2 pathway.

Some endogenous thiols, such as the redox couples glutathione/glutathione disulfide, cysteine/cystine and thioredoxin-reduced/thioredoxin-oxidized, are well known as markers of OS because of their antioxidant and cellular protective roles. Therefore, the paper by Paul and Snyder reviewed the role of the cysteamine, an aminothiol resultant from decarboxylation of amino acid cysteine, and its oxidized disulfide form cystamine in the progression of NDs through the modulation of different multiple targets, such as those from brain-derived neurotrophic factors and Nrf2 signaling pathways. The authors reported many evidences of the participation of cysteamine/cystamine and/or their metabolites in the reduction of OS and, consequently, promoting upregulation of cytoprotective effects in HD and other NDs.

The search for new small molecule bioactive compounds able to modulate receptors stimulated by lipid mediator sphingosine-1-phosphate (S1P) represents a great opportunity to discover drug candidates for NDs. One example is the agonist S1P receptor fingolimod, approved by FDA in 2010 as the first oral treatment for multiple sclerosis, but that also demonstrated ability to promote neuroprotection in animal models of PD. Stimulated by these previous results, Pépin et al. (2020) investigated the profile of compound SEW2871, a selective agonist of S1P receptors in animal models of PD. The obtained results clearly indicated that SEW2871 presented a neuroprotective activity similar to that displayed by fingolimod in 1-methyl-4-phenyl-1,2,3,6-tetrahydropyridine-induced mouse model of PD, being a promising candidate for treatment of this ND.

Last, but not least, the paper by Cassano et al. (2020) reviewed the therapeutic effects of cannabidiol, a non-psychoactive component of *Cannabis sativa*, highlighting not only its profile as a cannabinoid receptors antagonist, but also as a modulator of multiple signaling pathways and receptors, such as the *N*-methyl-D-aspartate. Thus, its ability to stimulate the production of important anti-inflammatory and antioxidant endogenous molecules makes this natural product an important drug candidate for treatment of different NDs.

For our concluding remarks, the contributions of this Research Topic indicated that, despite the morphological and biochemical differences among NDs, OS is recognized as a common component of these pathological conditions. In addition, many natural products belonging to different chemical classes and synthetic derivatives have been shown to be able to modulate OS by controlling ROS levels directly or indirectly by increasing the expression of antioxidant proteins, being important therapeutic alternatives for the treatment of NDs, possibly in combination with other therapies.

## Author Contributions

CF, MS, AT, and CV discussed and revised the contents of the paper. CF drafted the manuscript.

## Conflict of Interest

The authors declare that the research was conducted in the absence of any commercial or financial relationships that could be construed as a potential conflict of interest.

## References

[B1] Cassano T. Villani R. Pace L. Carbone A. Bukke V. N. Orkisz S. (2020). From Cannabis sativa to Cannabidiol: promising therapeutic candidate for the treatment of neurodegenerative diseases. Front. Pharmacol. 11, 124 10.3389/fphar.2020.00124 32210795 PMC7069528

[B2] Chen X. Guo C. Kong J. (2012). Oxidative stress in neurodegenerative diseases. Neural Regen. Res. 7 (5), 376–385. 10.3969/j.issn.1673-5374.2012.05.009 25774178 PMC4350122

[B3] Feigin V. L. Nichols E. Alam T. Bannick M. S. Beghi E. Blake N. (2019). Global, regional, and national burden of neurological disorders, 1990–2016: a systematic analysis for the Global Burden of Disease Study 2016. Lancet Neurol. 18 (5), 459–480. 10.1016/S1474-4422(18)30499-X 30879893 PMC6459001

[B4] Ghosh N. Ghosh R. Mandal S. C. (2011). Antioxidant protection: a promising therapeutic intervention in neurodegenerative disease. Free Radic. Res. 45 (8), 888–905. 10.3109/10715762.2011.574290 21615270

[B5] Golde T. E. (2009). The therapeutic importance of understanding mechanisms of neuronal cell death in neurodegenerative disease. Mol. Neurodegener. 4 (1), 8 10.1186/1750-1326-4-8 19193222 PMC2644308

[B6] Gribkoff V. K. Kaczmarek L. K. (2017). The need for new approaches in CNS drug discovery: why drugs have failed, and what can be done to improve outcomes. Neuropharmacology 120, 11–19. 10.1016/j.neuropharm.2016.03.021 26979921 PMC5820030

[B7] Huang B. Liu J. Fu S. Zhang Y. Li Y. He D. (2020). α-Cyperone attenuates H2O2-induced oxidative stress and apoptosis in SH-SY5Y cells via activation of Nrf2. Front. Pharmacol. 11, 281 10.3389/fphar.2020.00281 32322198 PMC7156596

[B8] Islam M. T. (2016). Oxidative stress and mitochondrial dysfunction-linked neurodegenerative disorders. Neurol. Res. 39 (1), 73–82. 10.1080/01616412.2016.1251711 27809706

[B9] Liu Z. Zhou T. Ziegler A. C. Dimitrion P. Zuo L. (2017). Oxidative stress in neurodegenerative diseases: from molecular mechanisms to clinical applications. Oxid. Med. Cell. Longev. 2017, 1–11. 10.1155/2017/2525967 PMC5529664 28785371

[B10] Martin J. B. (1999). Molecular basis of the neurodegenerative disorders. N. Engl. J. Med. 340 (25), 1970–1980. 10.1056/NEJM199906243402507 10379022

[B11] Mhillaj E. Cuomo V. Trabace L. Mancuso C. (2019). The heme oxygenase/biliverdin reductase system as effector of the neuroprotective outcomes of herb-based nutritional supplements. Front. Pharmacol. 10, 1298 10.3389/fphar.2019.01298 31780933 PMC6859463

[B12] Pépin É. Jalinier T. Lemieux G. L. Massicotte G. Cyr M. (2020). Sphingosine-1-phosphate receptors modulators decrease signs of neuroinflammation and prevent Parkinson’s disease symptoms in the 1-methyl-4-phenyl-1,2,3,6-tetrahydropyridine mouse model. Front. Pharmacol. 11, 77 10.3389/fphar.2020.00077 32153401 PMC7047735

[B13] Rekatsina M. Paladini A. Piroli A. Zis P. Pergolizzi J. V. Varrassi G. (2020). Pathophysiology and therapeutic perspectives of oxidative stress and neurodegenerative diseases: a narrative review. Adv. Ther. 37, 113–139. 10.1007/s12325-019-01148-5 PMC6979458 31782132

[B14] Santiago J. A. Bottero V. Potashkin J. A. (2017). Dissecting the molecular mechanisms of neurodegenerative diseases through network biology. Front. Aging Neurosci. 9, 166 10.3389/fnagi.2017.00166 28611656 PMC5446999

[B15] Sayre L. Smith M. Perry G. (2001). Chemistry and biochemistry of oxidative stress in neurodegenerative disease. Curr. Med. Chem. 8 (7), 721–738. 10.2174/0929867013372922 11375746

[B16] Schieber M. Chandel N. S. (2014). ROS function in redox signaling and oxidative stress. Curr. Biol. 24, R453–R462. 10.1016/j.cub.2014.03.034 24845678 PMC4055301

[B17] Seyhan A. A. (2019). Lost in translation: the valley of death across preclinical and clinical divide – identification of problems and overcoming obstacles. Trans. Med. Commun. 4 (1), 18 10.1186/s41231-019-0050-7

[B18] Singh A. Kukreti R. Saso L. Kukreti S. (2019). Oxidative stress: a key modulator in neurodegenerative diseases. Molecules 24, 1583 10.3390/molecules24081583 PMC6514564 31013638

[B19] Tan X. Yang Y. Xu J. Zhang P. Deng R. Mao Y. (2020). Luteolin exerts neuroprotection via modulation of the p62/Keap1/Nrf2 pathway in intracerebral hemorrhage. Front. Pharmacol. 10, 1551 10.3389/fphar.2019.01551 32038239 PMC6985769

[B20] Vadakkan K. I. (2016). Neurodegenerative disorders share common features of “loss of function” states of a proposed mechanism of nervous system functions. Biomed. Pharmacother. 83, 412–430. 10.1016/j.biopha.2016.06.042 27424323

[B21] Zhou Y. Wang X. Ying W. Wu D. Zhong P. (2019). Cryptotanshinone attenuates inflammatory response of microglial cells via the Nrf2/HO-1 pathway. Front. Neurosci. 13, 852 10.3389/fnins.2019.00852 31496930 PMC6712928

